# The influence of phosphorus source and the nature of nitrogen substrate on the biomass production and lipid accumulation in oleaginous *Mucoromycota* fungi

**DOI:** 10.1007/s00253-020-10821-7

**Published:** 2020-08-13

**Authors:** Simona Dzurendova, Boris Zimmermann, Valeria Tafintseva, Achim Kohler, Dag Ekeberg, Volha Shapaval

**Affiliations:** 1 Faculty of Science and Technology, Norwegian University of Life Sciences, Droebakveien 31, 1430 Aas, Norway; 2 Faculty of Chemistry, Biotechnology and Food Science, Norwegian University of Life Sciences, Christian Magnus Falsens vei 1, 1433 Aas, Norway

**Keywords:** Oleaginous fungi, Phosphorus, Nitrogen, Lipid profile, Micro-cultivation

## Abstract

**Abstract:**

Oleaginous filamentous fungi grown under the nitrogen limitation, accumulate high amounts of lipids in the form of triacylglycerides (TAGs) with fatty acid profiles similar to plant and fish oils. In this study, we investigate the effect of six phosphorus source concentrations combined with two types of nitrogen substrate (yeast extract and ammonium sulphate), on the biomass formation, lipid production, and fatty acid profile for nine oleaginous *Mucoromycota* fungi. The analysis of fatty acid profiles was performed by gas chromatography with flame ionization detector (GC-FID) and the lipid yield was estimated gravimetrically. Yeast extract could be used as both nitrogen and phosphorus source, without additional inorganic phosphorus supplementation. The use of inorganic nitrogen source (ammonium sulphate) requires strain-specific optimization of phosphorus source amount to obtain optimal lipid production regarding quantity and fatty acid profiles. Lipid production was decreased in ammonium sulphate-based media when phosphorus source was limited in all strains except for *Rhizopus stolonifer.* High phosphorus source concentration inhibited the growth of *Mortierella* fungi. The biomass (22 g/L) and lipid (14 g/L) yield of *Umbelopsis vinacea* was the highest among all the tested strains.

**Key points:**

*• The strain specific P requirements of Mucoromycota depend on the nature of N source.*

*• Yeast extract leads to consistent biomass and lipid yield and fatty acids profiles.*

*• Umbelopsis vinacea showed the highest biomass (22 g/L) and lipid (14 g/L) yield.*

*• High P source amounts inhibit the growth of Mortierella fungi.*

**Electronic supplementary material:**

The online version of this article (10.1007/s00253-020-10821-7) contains supplementary material, which is available to authorized users.

## Introduction

Unsaturated lipids are essential components in a human and animal nutrition and are traditionally obtained from fish and vegetable oils. Monounsaturated lipids are one of the major raw materials for lipid-based biofuels, which are nowadays mostly derived from vegetable and/or waste cooking oils. Recently, increased attention to the ocean protection and fishing regulations for avoiding the overfishing and preserving fish species highlighted the need to find alternative sources of essential unsaturated lipids (Pinheiro et al. 2018; Sala et al. 2018). Furthermore, on-going transition of the global economy towards renewable bioresources and the continuous discussion on the controversial usage of vegetable oils for biofuel vs food applications, created an increasing need for alternative sources of lipids (Correa et al. 2019; Meyer et al. 2020).

Oleaginous microbial biomass is considered as an alternative source of high- and low-value unsaturated lipids for food, feed, chemical industry, and lipid-based biofuels (Ratledge 2010). Oleaginous microorganisms, such as filamentous fungi, yeast and microalgae, are able to accumulate lipids up to 85% (w/w) of their total cell mass (Bharathiraja et al. 2017). Cellular oils are mainly produced in the form of free fatty acids, acylglycerols (mostly as triglycerides-TAGs) and other fatty acid-based lipids, that are stored in the globular organelles called lipid bodies. TAGs are generally considered as storage lipids. Depending on the fungal producer, accumulated lipids can be very similar to either vegetable oils, where saturated and monounsaturated fatty acids dominate, or to fish oils, where monounsaturated and polyunsaturated fatty acids dominate. Fatty acids derived from fungal lipids range from high-volume/low price to low-volume/high price. Examples for high-volume/low price fatty acids are monounsaturated fatty acids and saturated fatty acids that are used for the production of biodiesel, surfactants, soaps, resins, stabilizers, etc. On the other hand, high-price polyunsaturated fatty acids (ω3-PUFAs) may achieve high market value in pharmaceutical and food industry (van der Voort et al. 2017).

Oleaginous *Mucoromycota* fungi are considered as promising oleaginous microorganisms due to the relatively fast growth and high metabolic activity for utilizing both sugar- and lipid-based substrates. They are able to valorize a broad spectrum of low-cost substrates, including lignocellulose hydrolysates (Qiao et al. 2018; Subhash and Mohan 2015), sugar beet pulp (Ozsoy et al. 2015), wastewater (Bhanja et al. 2014), corncob waste liquor (Subhash and Mohan 2011), oil wastes (Mirbagheri et al. 2015), cheese whey permeate (Chan et al. 2018), and starch hydrolysates (Zhu et al. 2003).

In order to utilize low-cost substrates for the sustainable production of fungal lipids, there is a need to optimize their chemical composition. Carbon, nitrogen and phosphorus are the main components present in different low-cost substrates, and they are the key nutrients involved in the biomass growth and lipid accumulation in oleaginous microorganisms (Ratledge and Wynn 2002). Under the nitrogen-limiting condition, carbon is converted into TAGs which are stored in lipid bodies. Nitrogen is required for the proliferation and growth of fungal cells and as soon as it is depleted, the activity of isocitrate dehydrogenase is inhibited, and overproduced citrate is transported from mitochondria to the cytosol (Jiru and Abate 2014). Furthermore, ATP citrate lyase, which is a key enzyme of lipogenesis, cleaves the citrate into acetyl-CoA, which is reduced by the malic enzyme providing NADPH for fatty acid synthase. Thus, the backbone for fatty acids can be created (Akpinar-Bayizit 2014). Phosphorus is a part of phosphorylated molecules essential in lipid biosynthesis, such as energy transfer molecules adenosine mono-, di-, triphosphate (AMP, ADP, ATP), key lipogenesis enzyme ATP-citrate lyase, and reduced nicotinamide adenine dinucleotide phosphate (NADPH), which is directly involved in fatty acid synthesis as reductant. In addition, phosphorus is involved in the formation of lipid droplets, as it is the part of phospholipids of the lipid droplet membrane (Ratledge 2004).

Extensive number of studies focused on the understanding of the utilization of different carbon and nitrogen sources (Cortes and de Carvalho 2015; Evans and Ratledge 1983; Fakas et al. 2009; Heredia-Arroyo et al. 2011; Papanikolaou et al. 2007) and influence of different C/N ratios on the lipid accumulation in oleaginous microorganisms has been performed (Braunwald et al. 2013; Dyal et al. 2005; Economou et al. 2011; Evans and Ratledge 1984; Prasad et al. 2008; Ykema et al. 1988). The effect of phosphorus on the lipid production in algae has already been addressed (Chiriboga and Rorrer 2019; Esakkimuthu et al. 2016), and it was shown to be specie-dependent. In some cases, phosphorus starvation induced and enhanced the lipid accumulation (Feng et al. 2012; Roopnarain et al. 2014; Wu et al. 2013) while in other cases, it had the opposite effect (Li et al. 2014). Concerning oleaginous yeasts, phosphorus source limitation is beneficial for the lipid accumulation in nitrogen not-limited conditions (Huang et al. 2018; Wang et al. 2018; Wu et al. 2010). In case of filamentous fungi, the effect of phosphorus was investigated in connection to polyphosphate accumulation (Lima et al. 2003) or chitosan production (Safaei et al. 2016). To the authors knowledge, there is no study reported the investigation of the role of phosphorus on the lipid accumulation in oleaginous filamentous fungi.

Most of the reported studies used the traditional approach for triggering lipid accumulation by limiting either nitrogen or phosphorus nutrients when carbon is in excess. Our study is the first investigation of the influence of various phosphorus source concentrations under nitrogen-limiting conditions, when using different nitrogen sources. Two types of nitrogen sources were used—yeast extract (YE) and ammonium sulphate (AS). Yeast extract is a rich organic N-source, containing, in addition to 10% of nitrogen, approx. 2.5% of phosphorus, as well as a broad range of other macro- and micronutrients. Yeast extract was shown to be beneficial for biomass and lipid production for oleaginous *Mucoromycota* fungi (Dyal et al. 2005; Kosa et al. 2018b). Ammonium sulphate is a simple inorganic source of nitrogen, and it allows precise control of the chemical composition of all added nutrients.

The aim of the study was to investigate the influence of nitrogen source nature and the phosphorus source availability under nitrogen-limiting conditions on the biomass growth, lipid accumulation, and fatty acid profile of triacylglycerides for nine oleaginous *Mucoromycota* fungi, which were selected based on previously reported high-throughput screening study (Kosa et al. 2018b). High throughput micro-cultivation setup which employs the Duetz microtiter plates was used for presented screening (Duetz and Witholt 2001; Dzurendova et al. 2020; Kosa et al. 2017a; b; 2018b). A complete biochemical composition of the produced fungal biomass revealing the co-production potential has been assessed by Fourier transform infrared spectroscopy (FTIR-HTS), and the substrate consumption was monitored by FTIR-ATR (Dzurendova et al. 2020).

## Materials and methods

### Oleaginous *Mucoromycota* fungi

Nine oleaginous *Mucoromycota* fungi from the genera *Absidia*, *Amylomyces*, *Cunninghamella*, *Lichtheimia*, *Mortierella*, *Mucor*, *Rhizopus*, and *Umbelopsis* were used in the study (Table 1).

**Table 1:** List of the oleaginous *Mucoromycota* fungi used in the study

Family	Fungal strain name	Short name	Collection no.
*Cunninghamellaceae*	*Absidia glauca*	AGL	CCM^1^ 451
*Cunninghamellaceae*	*Cunninghamella blakesleeana*	CBL	CCM F705
*Cunninghamellaceae*	*Lichtheimia corymbifera*	LCO	CCM 8077
*Mortierellaceae*	*Mortierella alpina*	MAL	ATCC^2^ 32222
*Mortierellaceae*	*Mortierella hyalina*	MHY	VKM^3^ F1629
*Mucoraceae*	*Amylomyces rouxii*	ARO	CCM F220
*Mucoraceae*	*Mucor circinelloides*	MCI	VI^4^ 04473
*Mucoraceae*	*Rhizopus stolonifer*	RST	VKM F-400
*Umbelopsidaceae*	*Umbelopsis vinacea*	UVI	CCM F539

^1^Czech collection of Microorganisms (Brno, Czech Republic), ^2^American Type Culture Collection (Virginia, USA), ^3^All-Russian Collection of Microorganisms (Moscow, Russia), and ^4^Norwegian school of Veterinary Science (Oslo, Norway)

### Growth media and cultivation conditions

The cultivation of the fungi was performed in two steps: (1) cultivation on agar plates for a spore inoculum preparation, and (2) cultivation triggering lipid accumulation by using nitrogen-limited broth media with the different nitrogen sources (ammonium sulphate and yeast extract) and different amounts of inorganic phosphorus salts (Pi). For the preparation of the spore inoculum, malt extract agar (MEA) was used for all fungi with the exception of MAL, MHY, and UVI*,* which were cultivated on potato dextrose agar (PDA). MEA was prepared by dissolving 30 g of malt extract (Merck, Darmstadt, Germany), 5 g of peptone (Amresco, Solon, Ohio, USA), and 15 g of agar powder (Alfa Aesar, ThermoFischer, Kandel, Germany) in 1 L of distilled water and autoclaved at 115 °C for 15 min. PDA was prepared by dissolving 39 g of potato dextrose agar (VWR, Leuven, Belgium) in 1 L of distilled water and autoclaved at 115 °C for 15 min. Agar cultivations were performed for 7 days at 25 °C for all fungi except MAL and MHY, which were grown for 14 days due to the slower growth. Fungal spores were harvested with a bacteriological loop after the addition of 10 mL of sterile 0.9% NaCl solution.

The main components of the nitrogen-limited broth media were prepared according to the previously published studies on the screening of *Mucoromycota* fungi (Kavadia et al. 2001; Kosa et al. 2017a), with the following modifications (g/L): glucose 80, yeast extract 3, MgSO_4_·7H_2_O 1.5, CaCl_2_·2H_2_O 0.1, FeCl_3_·6H_2_O 0.008, ZnSO_4_·7H_2_O 0.001, CoSO_4_·7H_2_O 0.0001, CuSO_4_·5H_2_O 0.0001, and MnSO_4_·5H_2_O 0.0001. For the media with ammonium sulphate (AS) as a nitrogen source, yeast extract (YE) was replaced with 1.5 g/L of (NH_4_)_2_SO_4_ in order to keep the same C/N ratio. Broth media with ammonium sulphate contained 0.05 g/L thiamin hydrochloride and 0.02 mg/L biotin (Zeng et al. 2012). Different concentrations of phosphate salts, namely KH_2_PO_4_ and Na_2_HPO_4_, were added to the main components of nitrogen-limited broth media, as described in Table 2. 7 g L^−1^ KH_2_PO_4_ and 2 g L^−1^ Na_2_HPO_4_ were selected as a reference concentration values (Pi1) as those have been used in the previous studies (Kavadia et al. 2001; Kosa et al. 2017a, 2018b). The broth media contained higher (up to 8 × Pi1) and lower (down to ¼ × Pi1) amounts of phosphate salts in comparison to the reference value (Table 2). Two salts, KCl and NaCl, have been added in the corresponding concentrations to the broth media with the decreased amount of inorganic phosphorus, in order to have equal K^+^ and Na^+^ ions as in the reference condition (Pi1). Broth media were autoclaved for 15 min at 121 °C. The starting pH of the media was 6.0 ± 0.3, and pH of the culture supernatant after the growth was recorded (Table S1 in the Supplementary Material).

**Table 2:** The list of concentrations of phosphate salts in the nitrogen-limited broth media

Concentration labeling	KH_2_PO_4_ (g L^−1^)	Na_2_HPO_4_ (g L^−1^)
Pi8	56	16
Pi4	28	8
Pi2	14	4
Pi1	7	2
Pi0.5	3.5	1
Pi0.25	1.75	0.5

Cultivation in the nitrogen-limited broth media was performed in the Duetz-MTPS (Enzyscreen, Heemstede, Netherlands) (Kosa et al. 2017b, 2018a), consisting of 24-square polypropylene deep well microtiter plates, low evaporation sandwich covers, and extra high cover clamp system, which were mounted into the shaking incubator MAXQ 4000 (Thermo Scientific, Oslo, Norway). Seven milliliters of the sterile broth media was transferred into the autoclaved microtiter plates, and each well was inoculated with 50 μL of the spore suspension. Cultivations were performed for 7 days at 25 °C and 400 rpm agitation speed (1.9 cm circular orbit). Fungal strains MAL and MHY were cultivated for 14 days due to the slow growth.

The cultivation was performed in full factorial design in three independent biological replicates for each fungus, phosphorus source concentration, and nitrogen source, resulting in 324 samples. Biological replicates were prepared on a separate microtiter plates at different time points. For every biological replicate, fresh spore suspension was prepared. Two biological replicates were used for the extraction of lipids, while three biological replicates were used for evaluating the biomass production.

### Extraction of lipids and GC-FID analysis of fatty acid profile

Direct transesterification was performed according to the Lewis et al. (2000) with modifications. Two milliters screw-cap polypropylene (PP) tubes were filled with 30 ± 3 mg of freeze-dried biomass, 250 ± 30 mg of acid-washed glass beads, and 500 μL of methanol. Further, fungal biomass was disrupted in a tissue homogenizer (Bertin Technologies Percellys Evolution, Montigny-le-Bretonneux, France). The disrupted fungal biomass was transferred into glass reaction tubes by washing the PP tube with 2400 μL methanol–chloroform–hydrochloric acid solvent mixture (7.6:1:1 *v*/v). One milligram of C13:0 TAG internal standard in 100 μL of hexane was added to the glass reaction tube (100 μL from a 10.2 mg/mL glyceryl tritridecanoate (C_42_H_80_O_6_, C13:0 TAG (13:0/13:0/13:0), Sigma-Aldrich, St. Louis, Missouri, USA). Reaction tubes were incubated at 90 °C for 1 h, followed by cooling to room temperature and the addition of 1 mL distilled water. The fatty acid methyl esters (FAMEs) were extracted by the addition of 2 mL hexane–chloroform mixture (4:1 v/v) and 10 s vortex mixing. The reaction tubes were centrifuged at 3000*g* for 5 min at 4 °C and the upper hexane phase was collected in glass tubes. The extraction step was repeated three times for each sample. Subsequently, the solvent was evaporated under nitrogen at 30 °C, and FAMEs were dissolved in 1.5 mL of hexane containing 0.01% of butylated hydroxytoluene (BHT, Sigma-Aldrich, St. Louis, Missouri, USA) and a small amount of anhydrous sodium sulfate (to remove traces of water in the sample). Samples were mixed by vortexing, and finally, dissolved FAMEs were transferred to the GC vials.

Fatty acid profile analysis was performed using gas chromatography system with flame ionization detector (GC-FID) 7820A GC System, Agilent Technologies, controlled by Agilent OpenLAB software (Agilent Technologies, Santa Clara, California, USA). Agilent J&W GC column 121-2323, DB-23, 20 m length; 0.180 mm diameter; 0.20-μm film was used for the separation of FAMEs. One microliter of the sample was injected in the 30:1 split mode with the split flow 30 mL/min. The inlet heater temperature was set on 250 °C and helium was used as the carrier gas. The total runtime for one sample was 36 min with the following oven temperature increase: initial temperature 70 °C for 2 min, after 8 min to 150 °C with no hold time, 230 °C in 16 min with 5 min hold time, and 245 °C in 1 min with 4 min hold time. For identification and quantification of fatty acids, the C4–C24 FAME mixture (Supelco, St. Louis, USA) was used as an external standard, in addition to C13:0 TAG internal standard.

The total lipid yield was estimated gravimetrically. Hexane containing extracted lipids was evaporated under nitrogen at 30 °C and the residuals of the solvent were removed by drying in an oven overnight at 150 °C.

### Data analysis

Unscrambler X version 10.5.1 (CAMO Analytics, Oslo, Norway) and Orange data mining toolbox version 3.16 (University of Ljubljana, Slovenia) (Demšar et al. 2013; Toplak et al. 2017) were used for averaging the GC data and performing principal component analysis (PCA). Data for PCA were normalized. Matlab R2018a (The Mathworks Inc., Natick, USA) was used for the analysis of the influence of nitrogen source and phosphorus availability on the total biomass and lipid yield. For each fungal strain, an ANOVA model was established to calculate the variation in the data introduced by the different design factors such as N-source, Pi concentration, and N-Pi interaction. In ANOVA model, one represents the original data matrix as a sum of matrices corresponding to the experimental design factors. Each design matrix consists of means of rows corresponding to the levels of each design factor. The ANOVA model in this study contained three design factors: N-source, Pi concentration, the interaction of N-source and Pi level. All other variation was summarized in a matrix representing residual variation (Harrington et al. 2005)*.*

## Results

### The influence of the nitrogen source nature and phosphorus availability on the biomass and lipid yield

Two types of nitrogen (N) sources, organic yeast extract (YE) and inorganic ammonium sulphate (AS), and six concentrations of inorganic phosphorus salts (Pi) were used to study the influence of the nitrogen source nature and Pi substrate availability on the biomass production, lipid accumulation, and fatty acid profile of the accumulated TAGs in oleaginous *Mucoromycota* fungi. High glucose concentration (80 g/L) with low N substrate availability was used in order to induce lipid accumulation. The same glucose amount was used in our previous *Mucoromycota* studies (Kosa et al. 2017a; b; 2018b), and it was shown to be sufficient for cultivation lasting 12 days. HPLC analysis has shown residual glucose remaining after the cultivations. Thus, no glucose starvation, and, consequently, the utilization of produced fungal lipids as a carbon source was expected. The relative amount of residual glucose and phosphates in the culture supernatant was estimated by FTIR-ATR as published previously (Dzurendova et al. 2020). Both, phosphates and glucose were not fully utilized by fungi. UVI showed the highest glucose consumption, which corresponds to highest biomass production.

**Fig. 1:**
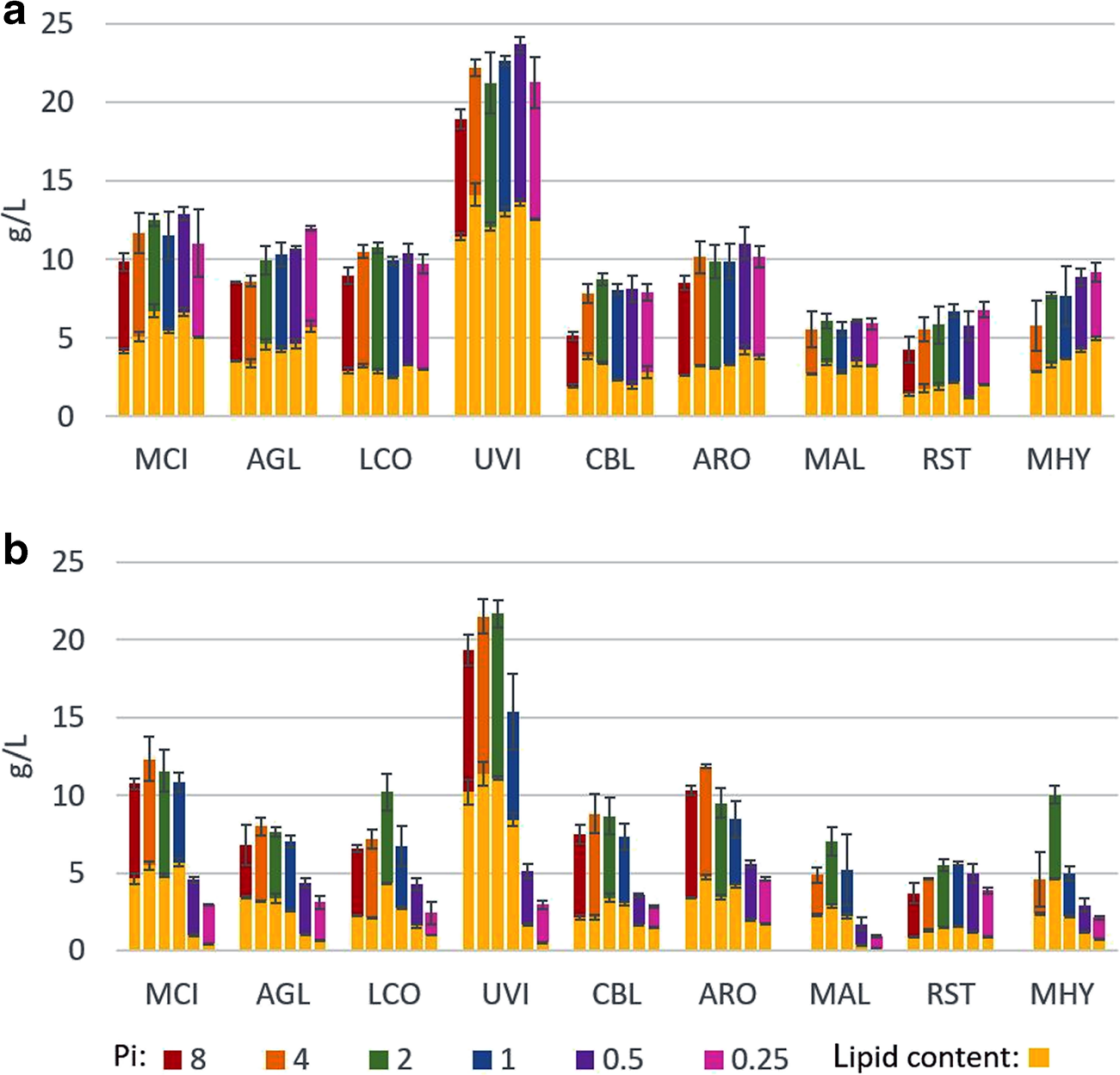
Biomass and lipid production of oleaginous *Mucoromycota* fungi grown in YE-Pi (**a**) and AS-Pi (**b**) based nitrogen-limited broth media

In the YE-Pi media, the highest biomass 18.92–23.67 g/L and lipid yield 11.46–14.13 g/L was observed for UVI (Fig. 1a). The obtained lipid content (57–63%) is in an agreement with the previously reported 51% (Zheng et al. 2012) and 66% (Meng et al. 2009)*.* While, the biomass production for UVI reported in our study was three times higher than in previously reported studies (7.1 g/L) (Zheng et al. 2012). Furthermore, a high biomass production was observed for MCI, AGL, LCO, and ARO in a range from 8.49 to 12.92 g/L with the lipid content from 2.52 to 6.7 g/L, where the highest biomass and lipid content was observed for MCI 12.92 g/L and 6.7 g/L, respectively (Fig. 1a), that is considerably higher than from previously reported studies (Zheng et al. 2012). Fungal strains CBL, MAL, MHY, and RST showed relatively low biomass production below 10 g/L, and lipid accumulation did not reach more than 3.82 g/L, where MAL had the lowest biomass 5.55–6.10 g/L and lipid 2.74–3.5 g/L yield (Fig. 1a).

High content of Pi source (Pi8) in the growth media led to the slight decrease in the growth and lipid accumulation in comparison to the moderate amounts of Pi source for all fungi, with completely inhibited growth for MAL and MHY*.* Moderate concentrations of inorganic phosphorus substrate (Pi4, Pi2, and Pi1) were optimal for the biomass and lipid production for the majority of the oleaginous *Mucoromycota* fungi when grown in AS-based media (Fig. 1b). Phosphorus source concentration Pi2 contributed to the highest biomass and lipid yield for LCO, UVI, MAL, and MHY. Pi4 was optimal for MCI, ARO, and AGL (Fig. 1b). Fungal strain RST showed an exceptional growth and lipid accumulation consistency, that was not affected by the Pi source availability and the nature of the nitrogen substrate (Fig. 1a, b).

As already mentioned, high phosphorus source availability showed inhibiting effect in YE-based media. On the other hand, AS-based media with high phosphorus source availability led to the increased biomass production for some fungi, such as CBL with AS-Pi8 (7.47 g/L) and Pi4 (8.83 g/L); ARO with AS-Pi8 (10.32 g/L) and AS-Pi4 (11.88 g/L), and MAL (7.03 g/L) and MHY (10.05 g/L) with AS-Pi2. In addition, lipid accumulation was increased for some fungi grown in AS-Pi media in comparison to the corresponding YE-Pi media, as for example, in the case of CBL with Pi1 (3.02 g/L), ARO Pi non-limited (3.40–4.20 g/L), and MHY with Pi2 (4.61 g/L) (Fig. 1a, b).

**Fig. 2:**
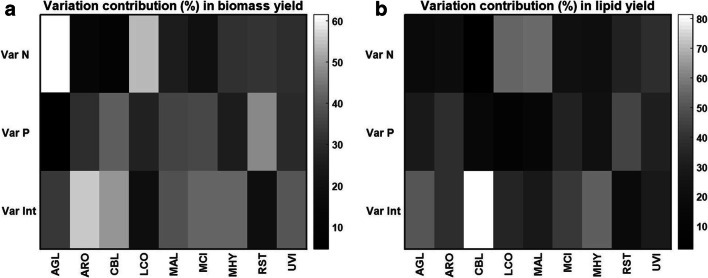
Variation contribution (%) from the changes in N, Pi, and N-Pi interaction on the biomass (g/L) (**a**) and lipid (% w/w) (**b**) yield. Variation contributions due to the changes in N and Pi alone are presented in the first two rows (Var N and Var P), whereas contribution from the N-Pi interaction (Int) is presented in the last row (Var Int)

ANOVA analysis was applied to perform overall study of the influence of the nitrogen source nature and the availability of inorganic phosphorus source on the variation in biomass and lipid yield (Fig. 2a, b). It was observed that the nature of the nitrogen source and phosphorus availability, both alone and in the interaction, introduce strain-specific and diverse changes in the biomass formation and lipid production in *Mucoromycota* fungi. The most substantial influence was observed from the interaction of both factors. Nature of the nitrogen source as a sole factor showed the biggest influence on the biomass production of AGL and LCO. Conversely, for ARO, CBL, and MCI, the nature of nitrogen substrate did not play the decisive role. The lipid production was significantly influenced by the nitrogen source for LCO, MAL, and UVI.

Variation in phosphorus source availability contributed to the most remarkable changes in the biomass and lipid production for RST. This was probably due to its extensive ability to store intracellular polyphosphates (Werner et al. 2007). The least phosphorus source contribution was observed for AGL. Lipid production for CBL was mostly affected by the interaction of both factors (N and Pi) (Fig. 2a, b).

### Fatty acid (FA) profile under different nitrogen sources and phosphorus substrate availability

**Fig. 3:**
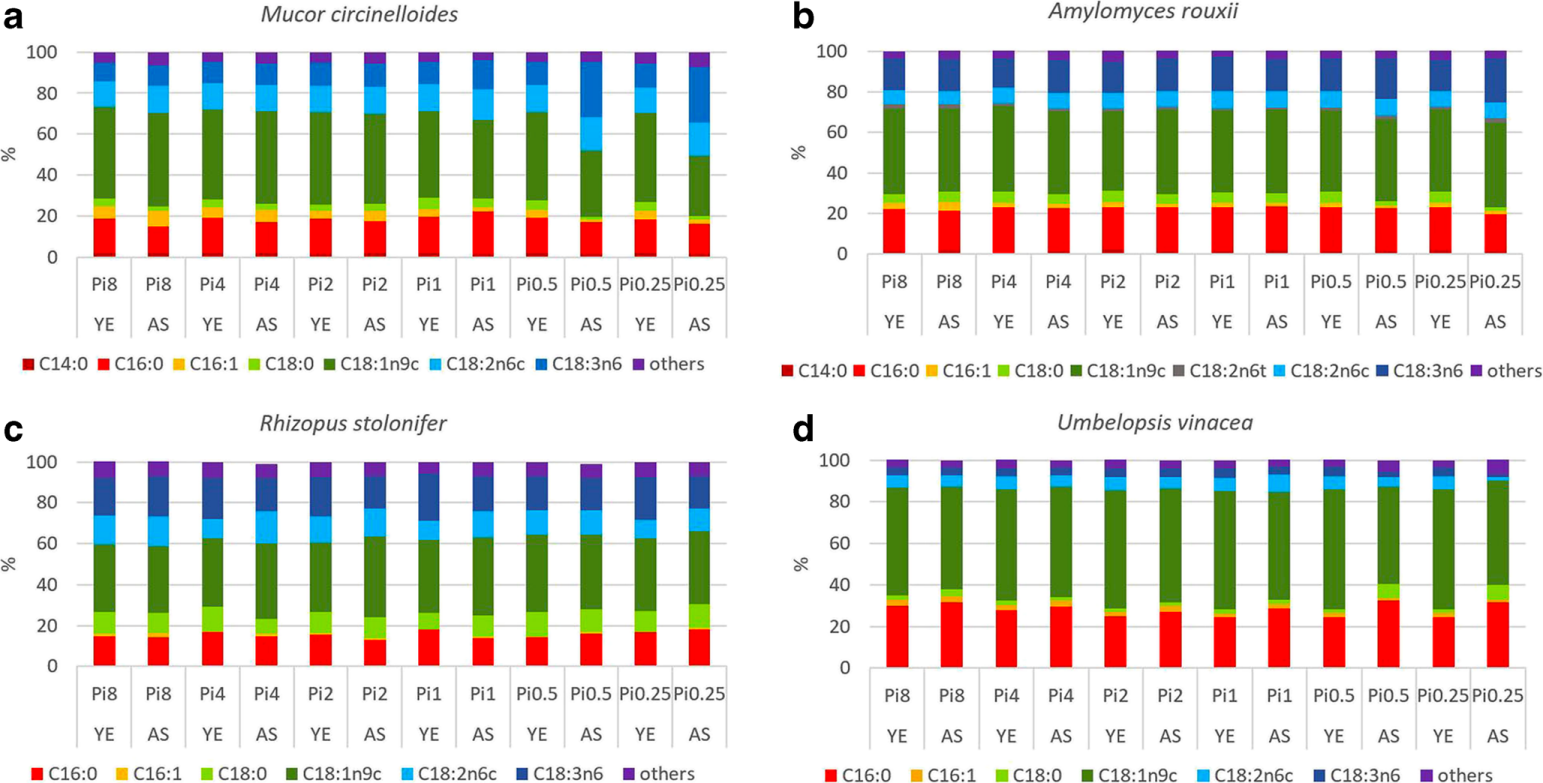
Fatty acid profiles *Mucoraceae* and *Umbelopsidaceae*. Fatty acids present in the amount higher than 1% are displayed; remaining fatty acids produced in a lower amount are summed up and presented as others

**Fig. 4:**
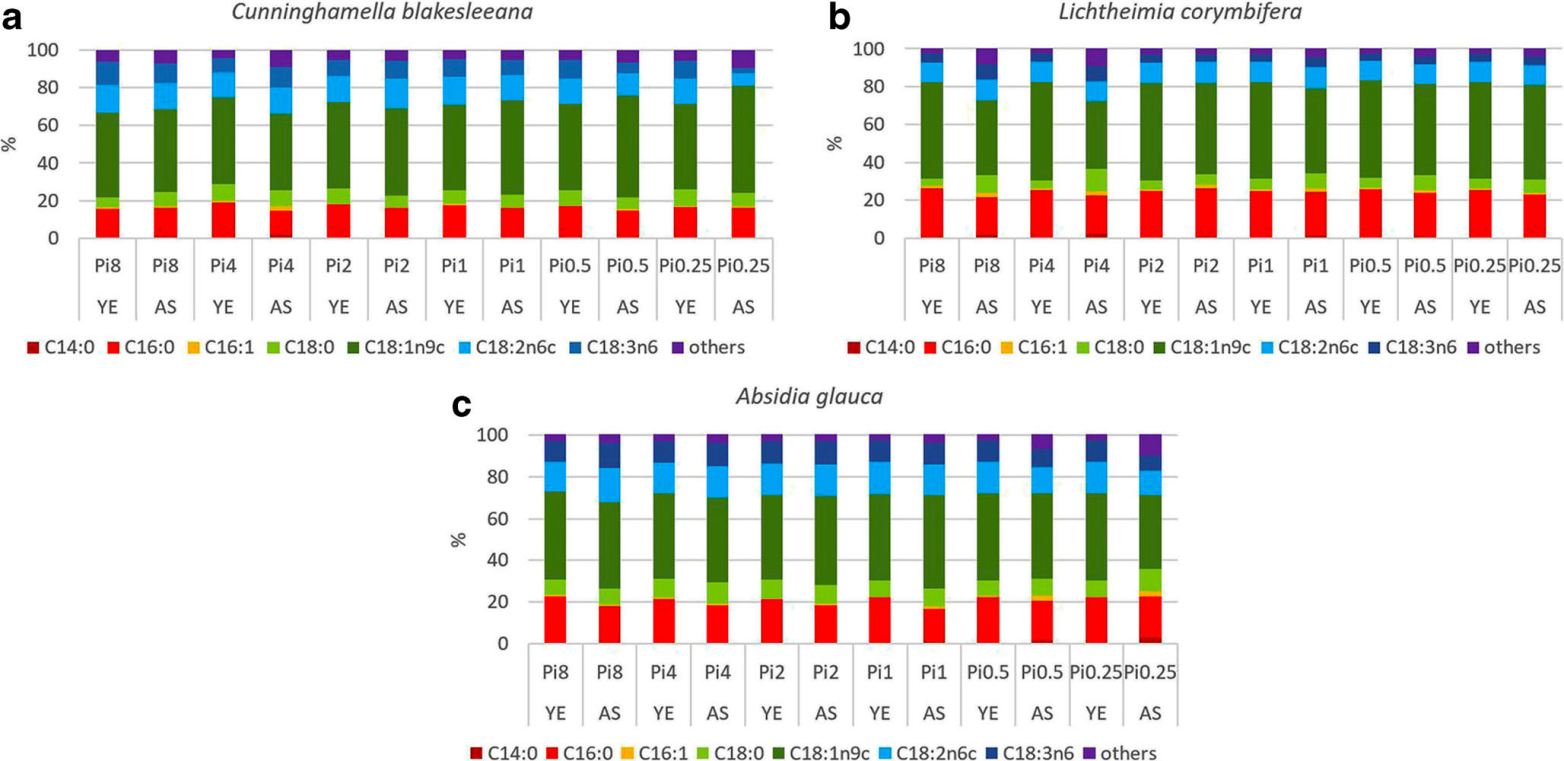
Fatty acid profile of *Cunninghamellaceae*. Fatty acids present in the amount higher than 1% are displayed; remaining fatty acids produced in a lower amount are summed up and presented as others

**Fig. 5:**
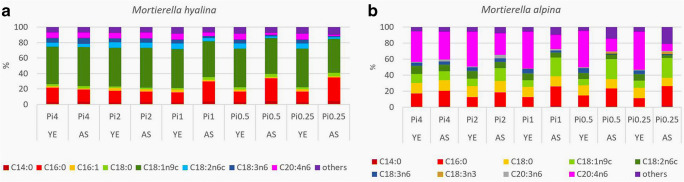
Fatty acid profiles of *Mortierellaceae*. Fatty acids present in the amount higher than 1% are displayed; remaining fatty acids produced in a lower amount are summed up and presented as others

The fatty acid profiles of *Mucoromycota* TAGs are dominated by the following fatty acids: myristic (C14:0), palmitic (C16:0), palmitolenic (C16:1), stearic (C18:0), oleic (C18:1n9), linoleic (C18:2n6), γ-linolenic (C18:3n6), and arachidonic (C20:4n6) acid. It was observed that fatty acid profiles are strain-specific while some similarities could be observed within the families *Mucoraceae* and *Umbelopsidaceae* (Fig. 3), *Cunninghamellaceae* (Fig. 4), and *Mortierellaceae* (Fig. 5). These results are in accordance with our previous study covering hundred *Mucoromycota* strains (Kosa et al. 2018b). Table S2 in the Supplementary Materials reports the detailed FA profiles.

The principal component analysis (PCA) of gas chromatography fatty acid profile data was performed to get an overview of the influence of phosphorus availability and the nature of the nitrogen source on the fatty acid profile of the TAGs, accumulated in *Mucoromycota* fungi (Fig. S1 in Supplementary Materials). The PCA scatter plot shows that fatty acid profile—sum of saturated (SAT), monounsaturated (MUFA), and polyunsaturated (PUFA) fatty acids—was relatively consistent when *Mucoromycota* fungi were grown in the YE-Pi media (A). The only exception was MAL, which under the high phosphorus source levels produced slightly more saturated TAGs (Fig. S1A in Supplementary Materials) and a decrease in arachidonic acid production (Pi4- 37.75%; Pi0.25–47%) was observed (Fig. 5).

The substantial variation in fatty acid profile of *Mucoromycota* TAGs was observed when fungi were grown in AS-Pi media (Fig. S1B in Supplementary Materials, Figs. 3, 4, and 5). Low phosphorus source availability (Pi0.5 and Pi0.25) led to acidic pH which induced clearly the most remarkable strain-specific changes in FA profiles for all the studied *Mucoromycota* fungi, except for LCO and RST (Fig. 3, Table S1 in Supplementary Materials). Thus, for *Mucoraceae* fungi, except RST*,* low Pi source amount resulted in the increase of the relative amount of the unsaturated fatty acids (γ-linolenic and linolenic) accompanied with the decrease in the amount of saturated (oleic and stearic) fatty acids (Fig. 3a, b). The opposite effect was observed for *Mortierellaceae* fungi, where amount of unsaturated fatty acids, specifically arachidonic fatty acid, decreased under the low phosphorus source availability (Fig. 5). Interestingly, MHY grown under the phosphorus limitation had a similar fatty acid profile as for the reference (Pi1) condition (Fig. 5). Fungi from the family *Cunninghamellaceae* showed diverse responses towards the low amounts of phosphorus source. For CBL, an increase in oleic acid up to 56.87% at Pi0.25 was observed (Fig. 4a). For AGL, a decrease in the content of oleic, linoleic, and γ-linolenic fatty acids, as well as an increase in stearic fatty acid was observed (Fig. 4b, Fig. 4c).

High (Pi8 and Pi4) phosphorus source availability did not have a significant influence on the fatty acid profile of TAGs accumulated in *Mucoraceae* fungi. For *Cunninghamellaceae*, except RST, high phosphorus source concentrations induced decrease in oleic acid accompanied with the increase in stearic acid (Fig. 4a, b). Thus, the difference in oleic fatty acid content for LCO and CBL was approximately 10% and 7%, respectively, in comparison to the reference phosphorus source condition (Pi1). Moreover, the relative content of stearic acid was doubled for LCO (Fig. 4). This is an indication that for these fungi high phosphorus source amount is possibly attenuating the activity of enzyme DS9, which is responsible for the desaturation of the bond at C9 position.

For ARO and RST, fatty acid profiles of the accumulated TAGs were not affected by the variation in inorganic phosphorus source and the type of nitrogen substrate. Further, interesting results were observed for UVI and LCO*.* These strains showed very similar lipid profile in YE-Pi media, while in AS-Pi media, phosphorus source availability affected these fungi in different ways. Increasing Pi source amount induced production of monounsaturated TAGs in UVI, whereas it led to more saturated lipids in LCO (Figs. 3, and 4)*.*

### Evaluating *Mucoromycota* lipids for biofuels application

Degree of unsaturation, or unsaturation index (UI), is an important parameter when evaluating the suitability of fatty acids for biofuel applications, and it is closely connected to the oxidation stability of lipids. The calculation of UI was performed for the TAGs of all *Mucoromycota* fungi with the exception of *Mortierella* strains, due to the fact that they produce relatively high amount of long-chain polyunsaturated fatty acids, which are as *tetraene*, not included in the calculation formula of UI. The unsaturation index was calculated as follows:

UI = [*Σ*(%monoene + 2 ×  % diene + 3 ×  % triene)]/100 (Sumner and Morgan 1969).

**Fig. 6:**
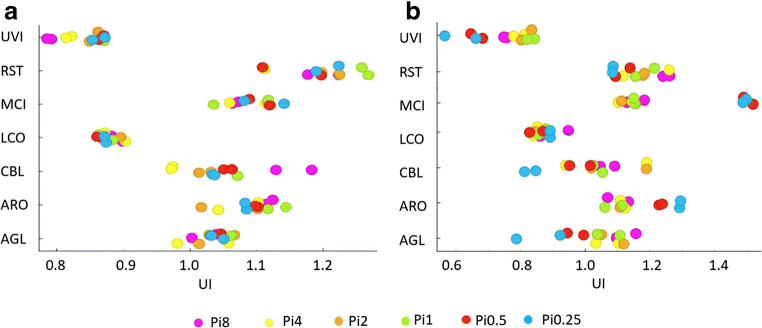
Unsaturation index of fungal TAGs grown in YE-Pi (**a**) and AS-Pi (**b**) media

It was observed that the UI of the produced in *Mucoromycota* TAGs, and respectively, the oxidation stability of the biofuel, increase with the limitation of phosphorus source in the growth medium. The UI was more stable for lipids produced by fungi grown in YE-Pi media, with the exception of UVI, where UI was lower with the increased phosphorus source availability. Limited availability of phosphorus source in the media with the ammonium sulphate resulted in a lower UI for MCI, ARO, CBL, AGL, and UVI (Fig. 6).

## Discussion

Fungi grown in YE-based nitrogen-limited broth media showed relatively stable biomass and lipid yield without significant changes depending on the level of inorganic phosphorus source (Pi) in comparison with ammonium sulphate (AS)-based media (Fig.1a, b). Yeast extract (YE) is a complex, nutrient rich N-source which is initially containing approx. 2.5% of organic phosphorus. Thus, the addition of moderate amounts of inorganic phosphorus substrate (Pi1, Pi2, and Pi4) had neglectable effects on the growth and lipid accumulation, while high (Pi8) and low (Pi0.5 and Pi0.25) Pi substrate concentrations influenced the fungal growth and lipid accumulation, as discussed further (Fig. 1a). A decrease in the biomass and lipid yield was observed for all *Mucoromycota* fungi when grown in the presence of high concentration of inorganic phosphorus source (Pi8) and YE as a nitrogen substrate (Fig. 1a). In addition, high Pi source amount (Pi8) was toxic for MAL and MHY strains and inhibited their growth completely. The opposite effect was observed when lower amount of Pi source (Pi 0.5 and Pi 0.25) was present in the media, the biomass and respectively lipid yield was increased for AGL, ARO, RST, and MHY (Fig. 1a). Thus, the observed results indicate that when using a rich organic nitrogen source such as yeast extract, the level of Pi source should not exceed Pi4 in order to achieve high biomass and lipid yield for *Mucoromycota* fungi.

The growth and lipid accumulation of oleaginous *Mucoromycota* fungi, grown in a nitrogen-limited broth-media with the inorganic nitrogen source ammonium sulphate (AS), were strongly influenced by the inorganic phosphorus source (Pi) availability. It was observed that fungal growth and lipid accumulation in the media with the low Pi source amounts (Pi0.5 and Pi0.25) were substantially lower than in the media with the moderate (Pi1, P2, and Pi4) and high Pi source concentrations (Pi8) (Fig. 1b). Inorganic phosphate salts play buffering role in the growth media, and their low levels led to the decrease of pH (Table S1 in Supplementary Material). Low pH as the consequence of low Pi source therefore negatively affected the growth and lipid accumulation. When decreased amount of phosphate salts (Pi0.5 and Pi0.25) was combined with YE, the buffering function of Pi was substituted by the buffering capacity of YE (Dzurendova et al. 2020). The major advantage of Duetz micro-cultivation setup is the miniaturization of culture volume, which allows high throughput screening of many different media and strains at the same time. Unfortunately, these can only be performed in a set-up with only start-end pH measurement without continuous pH tuning available. Bioreactor cultivation under pH-controlled conditions would shed light on the effect of low Pi concentrations when using AS on the biomass and lipid production, excluding the factor of low pH.

Our study showed that several *Mucoromycota* fungi have relatively low biomass production under lipogenesis conditions. Thus, the results of low biomass for *Rhizopus (oryzae)* and CBL were in accordance with the previously performed study by Janakiraman (2014). Furthermore, the biomass and lipid production of RST were quite low, approx. 5 g/L and 2 g/L, respectively. There are several strategies for the optimization of the biomass production, such as increasing the nitrogen substrate content in the media, addition of stimulators, and/or using different cultivation temperatures. For example, biomass yield and lipid accumulation for *Mortierella* species could be improved by supplementation with soy flour, vegetable oils, temperature switch, and fed-batch cultivation (Singh and Ward 1997). However, those were not tested since they were outside the scope of this study.

The fatty acid profile of fungal lipids is dependent on the growth phase, which was affected by the low pH caused by low Pi source availability in the AS-Pi media. When using above described transesterification method, fatty acids present in different types of lipids (mono-, di-, triglycerides; phospholipids, and free fatty acids) are turned into FAMEs. Our previous studies and reference literature support the fact that majority of lipids present in studied *Mucoromycota* are in the form of TAGs (Forfang et al. 2017; Ratledge 2010). Therefore, the majority of FAMEs obtained after transesterification originate from TAGs. Furthermore, when assessing lipids for biodiesel application, lipids are converted into FAMEs; therefore, the used transesterification protocol was suitable from this point of view. It was observed that low Pi source availability caused changes in the content of stearic, oleic, linoleic, γ-linolenic, and arachidonic acids. Thus, low Pi source concentrations have possibly affected the activity of enzymes DS9, DS12, EL, DS12, and DS6 (Fig. S2 in Supplementary Materials). It can be noted, that based on the fatty acid profiles, the activity of enzymes DS9, DS6, and DS12 seemed inhibited in some cases while for others, it was enhanced under the low Pi source availability. Interestingly, low amount of Pi substrate possibly inhibited the activity of desaturases (DS6, EL, and DS5), resulting in the decrease of polyunsaturated fatty acids. The importance of phosphorus source availability on the activity of desaturase enzymes could be revealed by the increased unsaturation of fatty acids in *Mortierella* strains under the moderate (Pi2) and high Pi (Pi4) source concentrations in AS-based media (Fig. 5). The same Pi source amounts in YE-based media caused decrease in the unsaturation, indicating that *Mortierellaceae* fungi would require a careful optimization of phosphorus source content in the media*.* Detailed enzymatic study would be needed to confirm the effect of Pi on the lipogenesis enzymes.

Due to the fact that single-cell oil-based biofuels are one of the rapidly growing biofuels sector demanding alternative source of lipids, we performed an evaluation of *Mucoromycota* lipids for possible biofuels application. It is well known, that the more double bonds are present in a fatty acid, the more it is prone towards the oxidation (Yaakob et al. 2014). While, some monounsaturated fatty acids, as for example, oleic acid, have been reported for being stable towards the oxidation (Hernandez 2016); polyunsaturated fatty acids, which are often produced by *Mucoromycota* fungi, are rapidly oxidizing molecules and therefore need to be avoided and separated from the lipids subjected to the production of lipid-based biofuels. Thus, the lower the UI of the produced fatty acids, the more suitable they are for the production of lipid-based biofuels. Among the studied *Mucoromycota* fungi, strains UVI, LCO, and CBL showed the most suitable UI of lipids for the lipid-based biofuels application. For strains producing TAGs containing increased amount of linolenic acid—ARO, RST, AGL, and MCI, it would possibly require the addition of antioxidants (Botella et al. 2014) when producing biofuels*.*

To conclude, yeast extract could be considered as a suitable organic N source requiring from very limited to no phosphorus substrate addition for obtaining consistent biomass and lipid yield and fatty acid profile. When inorganic nitrogen source ammonium sulphate was used, it required strain-specific optimization of phosphorus source concentration to achieve optimal biomass and lipid production as well as fatty acid profile. Low Pi source availability in AS-Pi media resulted in low pH which negatively affected the fungal growth. Considering the buffering capacity and the cost of yeast extract and ammonium sulphate, the economical sustainability of these substrates needs to be carefully evaluated. The price is dependent on required quality and amount. The price for yeast extract for use in microbial growth medium is 198 USD per kg (Sigma Aldrich, St. Louis, Missouri, USA; molecular biology grade), while the cost of ammonium sulphate is considerably lower—74 USD per kg (Sigma Aldrich, St. Louis, Missouri, USA; ReagentPlus grade, ≥ 99.0%).

We showed that phosphorus source influence on the biomass yield, lipid product, and fatty acid profile is strain-specific, and both low and/or high phosphorus source availability can have beneficial effects. Among the tested *Mucoromycota* fungi, interesting findings were observed for (i) UVI which showed extraordinary high biomass and lipid yield (22 g/L and 63.55%) at relatively high phosphorus source amount; (ii) RST showed an obvious advantage in managing the acidic pH caused by phosphorus source deficiency, since its growth, lipid accumulation, and fatty acid profile did not change under different phosphorus source amounts; (iii) MAL and MHY showed high sensitivity to the high levels of phosphorus substrate, while moderate amounts resulted in the increase of the lipid accumulation and unsaturation.

## Electronic supplementary material

ESM 1 (PDF 514 kb)

## Data Availability

The datasets generated and/or analyzed during the current study are available in the manuscript and its supplementary materials.
