# Supplementary material for: The influence of phosphorus source and the nature of nitrogen substrate on the biomass production and lipid accumulation in oleaginous Mucoromycota fungi
Source: Appl Microbiol Biotechnol. 2020 Aug 13;104(18):8065–76. doi: 10.1007/s00253-020-10821-7 (PMC7447667; doi:10.1007/s00253-020-10821-7)
Supplement: Supplementary file 1 — (PDF 514 kb) [file 253_2020_10821_MOESM1_ESM.pdf]

Applied Microbiology and Biotechnology

The influence of phosphorus source and the nature of nitrogen substrate on the biomass production and lipid accumulation in oleaginous *Mucoromycota* fungi

Simona Dzurendova[1*], Boris Zimmermann[1], Valeria Tafintseva[1], Achim Kohler[1], Dag Ekeberg[2], Volha Shapaval[1]

[1]Norwegian University of Life Sciences, Faculty of Science and Technology,

Droebakveien 31, 1430 Aas, Norway

[2]Norwegian University of Life Sciences, Faculty of Chemistry, Biotechnology and Food Science, Christian Magnus Falsens vei 1, 1433 Aas, Norway

*Correspondence:

Simona Dzurendova

email: simona.dzurendova@gmail.com; simona.dzurendova@nmbu.no

phone: +47 94785832

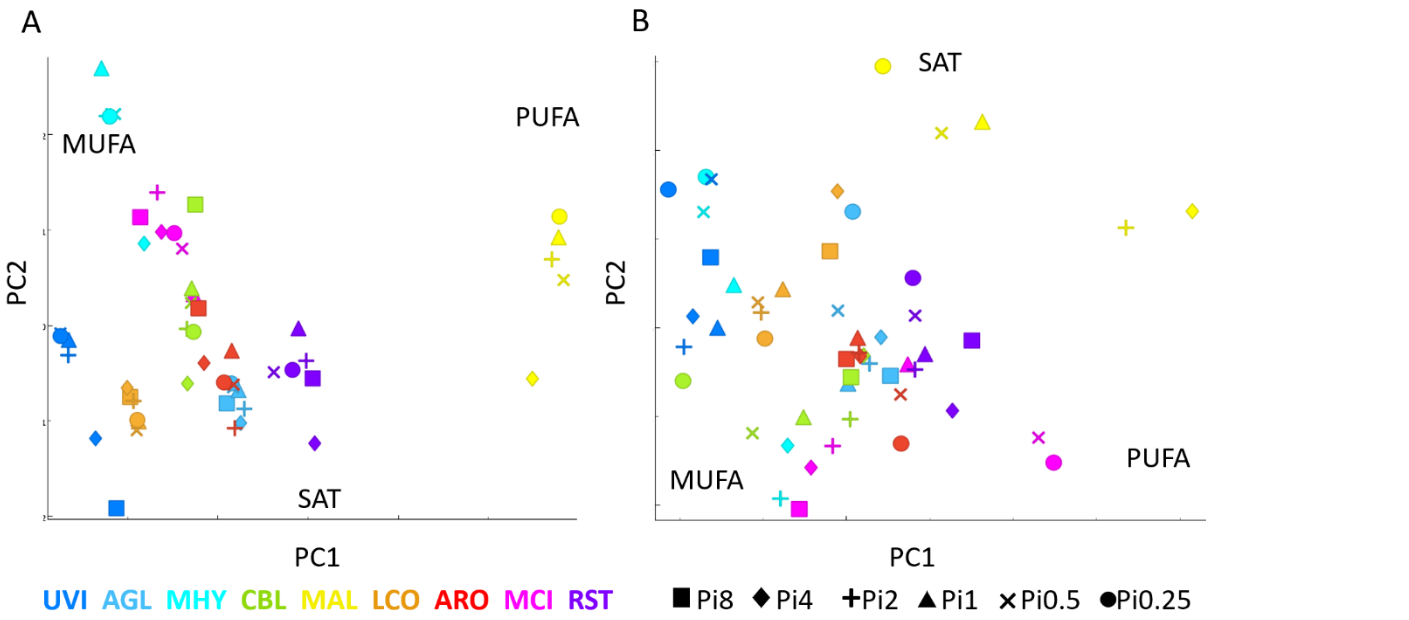

Fig. S1: PCA scatter plot of fatty acid profile of the accumulated TAGs in *Mucoromycota* fungi grown in the presence of different phosphorus amounts in the growth media with YE (A) and AS (B) as nitrogen source.

Table S1: Average pH of culture supernatant

**Average pH**		**Pi 8**	**Pi 4**	**Pi 2**	**Pi 1**	**Pi 0.5**	**Pi 0.25**
**MCI**	YE	5.91	5.98	5.95	5.97	5.59	4.30
	AS	5.55	5.38	4.34	2.91	2.34	2.21
**AGL**	YE	4.59	3.98	3.72	3.61	3.49	3.67
	AS	4.41	3.91	3.65	3.05	2.50	2.28
**LCO**	YE	5.54	5.59	5.56	5.54	5.33	5.25
	AS	5.61	5.46	4.59	3.03	2.50	2.41
**UVI**	YE	5.42	5.46	5.44	5.49	5.37	5.26
	AS	5.21	4.76	3.72	2.26	1.78	1.72
**CBL**	YE	5.72	5.56	5.48	5.19	4.78	4.35
	AS	5.35	4.77	3.96	2.52	2.07	2.94
**ARO**	YE	5.39	5.32	5.06	4.58	3.95	3.57
	AS	5.42	5.17	4.36	2.40	1.81	1.69
**MAL**	YE	6.00	5.55	5.59	5.72	5.61	5.51
	AS	5.80	5.49	4.36	3.08	2.97	2.95
**RST**	YE	5.19	3.97	3.74	3.90	3.26	4.41
	AS	5.02	3.87	3.01	2.32	1.86	1.66
**MHY**	YE	5.31	5.16	5.37	5.52	5.05	5.21
	AS	5.29	4.91	4.36	2.51	2.35	3.49

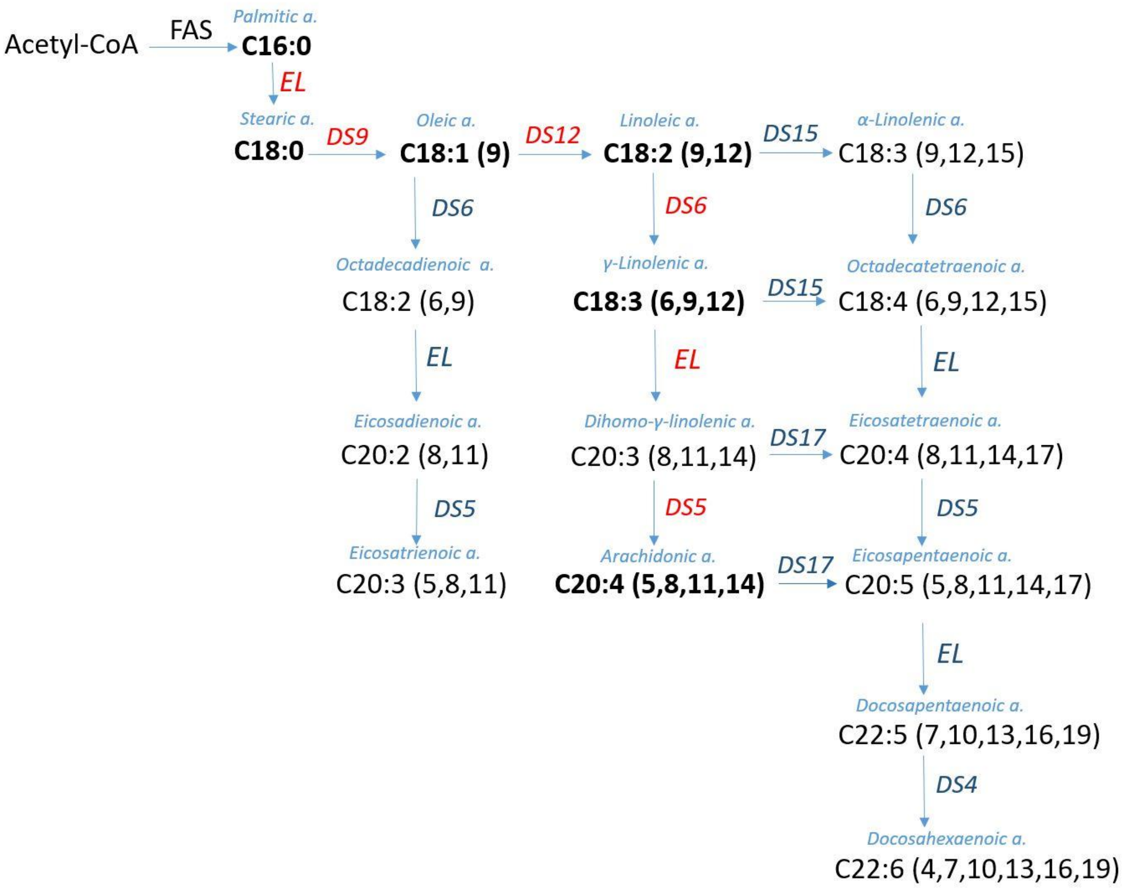

Fig. S2: Fatty acid synthesis in oleaginous microorganisms, adapted from (Ratledge and Wynn 2002). Fatty acids produced by strains used in the study are marked in bold. FAS- fatty acid synthase; DS- desaturase; EL- elongase. Enzymes affected by Pi variation are marked red.

Ratledge C, Wynn JP (2002) The biochemistry and molecular biology of lipid accumulation in oleaginous microorganisms Adv Appl Microbiol 51:1-52

Table S2: Fatty acid profiles

			C14:0	C16:0	C16:1	C18:0	C18:1n9c	C18:2n6t	C18:2n6c	C18:3n6	C20:4n6
**MCI**	**YE**	**Pi8**	2.05	16.67	6.03	3.53	45.13	0.75	12.22	9.09	0.04
**MCI**	**YE**	**Pi4**	1.91	17.17	4.94	3.98	44.23	0.66	12.84	10.10	0.04
**MCI**	**YE**	**Pi2**	1.74	16.90	4.07	2.77	45.37	0.61	12.96	10.95	0.05
**MCI**	**YE**	**Pi1**	1.88	17.74	3.69	5.61	42.28	0.52	13.23	10.70	0.04
**MCI**	**YE**	**Pi0.5**	1.98	17.08	3.99	4.66	43.18	0.62	13.10	11.22	0.06
**MCI**	**YE**	**Pi0.25**	2.02	16.39	4.16	4.26	43.53	0.74	12.59	11.38	0.02
**MCI**	**AS**	**Pi8**	1.97	12.89	7.73	2.05	45.71	0.86	13.08	10.04	0.03
**MCI**	**AS**	**Pi4**	1.88	15.05	6.10	2.88	45.34	0.68	12.98	10.23	0.07
**MCI**	**AS**	**Pi2**	1.96	15.47	4.94	3.51	44.10	0.67	13.19	11.32	0.06
**MCI**	**AS**	**Pi1**	1.52	20.71	1.82	4.41	38.37	0.28	15.09	14.24	0.06
**MCI**	**AS**	**Pi0.5**	1.63	15.49	0.92	1.40	32.63	0.68	16.06	27.08	0.07
**MCI**	**AS**	**Pi0.25**	1.67	14.59	2.06	1.51	29.79	0.75	16.07	26.92	0.14
**AGL**	**YE**	**Pi8**	0.55	22.16	0.73	7.36	42.32	0.11	13.95	9.98	0.02
**AGL**	**YE**	**Pi4**	0.52	21.10	0.65	9.04	40.91	0.13	14.57	9.96	0.02
**AGL**	**YE**	**Pi2**	0.48	20.87	0.59	8.97	40.60	0.12	14.59	10.75	0.03
**AGL**	**YE**	**Pi1**	0.51	21.74	0.58	7.64	41.34	0.10	15.08	10.42	0.02
**AGL**	**YE**	**Pi0.5**	0.54	21.81	0.60	7.47	41.83	0.10	14.86	10.15	0.02
**AGL**	**YE**	**Pi0.25**	0.54	21.64	0.58	7.53	42.07	0.10	14.90	10.17	0.02
**AGL**	**AS**	**Pi8**	0.39	17.72	0.75	7.81	41.43	0.25	15.89	12.26	0.04
**AGL**	**AS**	**Pi4**	0.36	18.23	0.71	10.08	40.57	0.23	14.89	11.36	0.04
**AGL**	**AS**	**Pi2**	0.42	18.15	0.76	8.82	42.73	0.24	14.79	11.29	0.03
**AGL**	**AS**	**Pi1**	0.90	15.81	1.32	8.59	44.68	0.42	14.42	10.18	0.02
**AGL**	**AS**	**Pi0.5**	1.98	18.61	2.62	8.06	40.86	0.82	12.49	8.49	0.09
**AGL**	**AS**	**Pi0.25**	2.93	19.67	2.52	10.66	35.53	0.74	11.67	6.91	0.06
**MHY**	**YE**	**Pi4**	2.76	18.88	1.92	2.68	48.24	0.14	5.09	6.07	7.50
**MHY**	**YE**	**Pi2**	2.53	14.93	3.58	2.01	50.31	0.09	6.80	5.02	7.31
**MHY**	**YE**	**Pi1**	2.58	12.93	3.43	2.04	50.74	0.06	7.25	4.81	7.67
**MHY**	**YE**	**Pi0.5**	2.63	13.88	2.69	2.71	50.10	0.06	6.81	4.99	7.73
**MHY**	**YE**	**Pi0.25**	2.70	14.05	2.83	2.46	50.33	0.07	6.54	4.91	7.44
**MHY**	**AS**	**Pi4**	3.24	15.71	1.49	3.37	50.55	0.26	3.89	7.69	7.19
**MHY**	**AS**	**Pi2**	2.89	13.74	3.15	2.03	51.47	0.08	6.36	5.90	7.21
**MHY**	**AS**	**Pi1**	3.78	25.94	1.97	3.43	46.94	0.14	3.64	2.79	4.68
**MHY**	**AS**	**Pi0.5**	4.37	29.13	1.62	4.31	46.29	0.18	2.34	1.62	2.62
**MHY**	**AS**	**Pi0.25**	4.21	30.28	1.78	4.61	44.02	0.10	1.93	1.39	1.86
**LCO**	**YE**	**Pi8**	0.71	25.44	1.72	3.62	51.07	0.06	10.11	4.86	0.02
**LCO**	**YE**	**Pi4**	0.61	24.55	1.29	4.24	51.88	0.06	10.64	4.33	0.02
**LCO**	**YE**	**Pi2**	0.57	24.19	1.05	4.89	51.37	0.05	10.88	4.21	0.02
**LCO**	**YE**	**Pi1**	0.54	24.56	0.96	5.25	50.92	0.06	10.75	4.14	0.02
**LCO**	**YE**	**Pi0.5**	0.59	25.11	1.03	5.26	51.16	0.06	10.49	4.00	0.03
**LCO**	**YE**	**Pi0.25**	0.56	24.76	0.98	5.15	51.11	0.06	10.65	4.17	0.03
**LCO**	**AS**	**Pi8**	2.06	19.53	2.36	9.56	39.20	0.27	10.84	8.24	0.05
**LCO**	**AS**	**Pi4**	2.53	20.11	2.18	11.62	36.20	0.26	10.31	7.82	0.04
**LCO**	**AS**	**Pi2**	0.77	25.75	1.68	5.65	48.10	0.07	11.25	3.97	0.04
**LCO**	**AS**	**Pi1**	1.31	22.93	2.25	7.79	44.69	0.18	11.20	5.15	0.03
**LCO**	**AS**	**Pi0.5**	0.89	23.23	1.39	7.74	48.20	0.16	10.24	4.45	0.04
**LCO**	**AS**	**Pi0.25**	0.37	22.69	0.95	6.95	49.77	0.11	10.64	4.82	0.07

			C14:0	C16:0	C16:1	C18:0	C18:1n9c	C18:2n6t	C18:2n6c	C18:3n6	C20:4n6
**CBL**	**YE**	**Pi8**	0.49	15.09	0.72	5.55	45.06	0.76	14.41	12.49	0.08
**CBL**	**YE**	**Pi4**	0.64	18.29	0.76	9.28	46.03	0.40	13.29	7.25	0.05
**CBL**	**YE**	**Pi2**	0.57	17.24	0.64	7.97	45.95	0.51	13.69	8.48	0.07
**CBL**	**YE**	**Pi1**	0.61	17.09	0.58	7.16	45.73	0.55	14.37	9.59	0.04
**CBL**	**YE**	**Pi0.5**	0.59	16.33	0.54	8.11	45.85	0.62	13.49	9.84	0.03
**CBL**	**YE**	**Pi0.25**	0.59	16.06	0.49	9.04	45.49	0.63	12.91	9.55	0.06
**CBL**	**AS**	**Pi8**	1.09	14.78	1.02	7.59	44.23	0.72	13.53	10.50	0.05
**CBL**	**AS**	**Pi4**	2.02	12.57	2.33	8.85	40.53	0.82	13.69	10.79	0.03
**CBL**	**AS**	**Pi2**	0.60	15.26	0.67	6.32	46.42	0.70	15.48	9.73	0.04
**CBL**	**AS**	**Pi1**	0.49	15.47	0.58	6.78	50.09	0.60	13.41	7.87	0.04
**CBL**	**AS**	**Pi0.5**	0.42	14.43	0.84	5.98	54.21	0.47	11.57	5.79	0.03
**CBL**	**AS**	**Pi0.25**	0.45	15.71	0.91	6.94	56.87	0.37	6.68	2.80	0.09
**UVI**	**YE**	**Pi8**	0.77	30.21	2.57	2.39	51.60	0.12	6.05	3.79	0.04
**UVI**	**YE**	**Pi4**	0.74	27.73	2.59	2.20	53.57	0.16	6.16	3.95	0.04
**UVI**	**YE**	**Pi2**	0.68	24.89	2.21	1.81	56.77	0.14	6.25	4.18	0.03
**UVI**	**YE**	**Pi1**	0.69	24.40	2.05	1.85	56.80	0.15	6.45	4.54	0.03
**UVI**	**YE**	**Pi0.5**	0.69	24.49	2.12	1.73	57.75	0.15	6.25	4.34	0.03
**UVI**	**YE**	**Pi0.25**	0.72	24.60	2.25	1.63	57.49	0.16	6.12	4.27	0.03
**UVI**	**AS**	**Pi8**	0.84	31.82	2.67	3.64	48.96	0.15	5.78	3.68	0.04
**UVI**	**AS**	**Pi4**	0.82	29.51	2.88	2.05	52.56	0.22	5.64	3.84	0.03
**UVI**	**AS**	**Pi2**	0.82	27.25	2.71	1.78	54.62	0.21	5.49	4.05	0.03
**UVI**	**AS**	**Pi1**	0.82	28.81	2.11	1.94	51.92	0.11	8.17	3.93	0.04
**UVI**	**AS**	**Pi0.5**	0.71	32.69	1.30	6.60	46.61	0.12	4.77	2.53	0.04
**UVI**	**AS**	**Pi0.25**	0.79	31.66	1.48	6.90	50.04	0.51	1.66	1.28	0.10
**RST**	**YE**	**Pi8**	0.73	14.88	1.16	10.82	32.91	0.49	14.07	18.22	0.07
**RST**	**YE**	**Pi4**	0.63	16.97	0.51	11.92	33.34	0.99	9.39	20.12	0.06
**RST**	**YE**	**Pi2**	0.66	15.77	0.65	10.33	33.93	0.67	12.60	19.43	0.03
**RST**	**YE**	**Pi1**	0.61	18.06	0.22	8.11	35.41	1.02	9.34	22.90	0.02
**RST**	**YE**	**Pi0.5**	0.61	14.11	0.69	11.97	37.49	0.63	12.10	16.79	0.02
**RST**	**YE**	**Pi0.25**	0.58	16.74	0.30	9.89	35.74	1.07	8.92	21.00	0.07
**RST**	**AS**	**Pi8**	0.92	14.47	1.82	10.05	32.54	0.40	14.38	19.85	0.05
**RST**	**AS**	**Pi4**	0.59	11.90	0.99	7.33	35.51	0.38	15.55	16.27	0.07
**RST**	**AS**	**Pi2**	0.60	13.01	0.76	10.23	39.28	0.41	13.87	16.01	0.04
**RST**	**AS**	**Pi1**	0.62	14.03	0.70	10.42	37.86	0.52	12.98	17.18	0.08
**RST**	**AS**	**Pi0.5**	0.69	15.95	0.83	11.08	36.52	0.49	11.92	15.88	0.04
**RST**	**AS**	**Pi0.25**	0.76	18.14	0.89	11.42	35.52	0.47	11.39	15.68	0.07
**ARO**	**YE**	**Pi8**	1.54	20.59	3.18	4.15	42.57	1.83	7.22	15.54	0.05
**ARO**	**YE**	**Pi4**	0.69	22.27	2.45	5.23	42.65	1.24	7.73	14.30	0.06
**ARO**	**YE**	**Pi2**	2.20	20.91	2.36	5.80	39.37	1.31	7.71	14.82	0.06
**ARO**	**YE**	**Pi1**	1.69	21.43	2.13	5.20	40.33	1.26	8.42	16.67	0.02
**ARO**	**YE**	**Pi0.5**	1.52	21.73	2.00	5.57	40.08	1.21	8.24	15.88	0.06
**ARO**	**YE**	**Pi0.25**	1.77	21.29	2.32	5.26	40.63	1.33	8.05	14.97	0.06
**ARO**	**AS**	**Pi8**	2.10	19.34	4.06	5.48	40.69	2.15	6.54	15.53	0.05
**ARO**	**AS**	**Pi4**	1.55	20.91	2.40	4.70	40.97	1.41	7.62	16.20	0.04
**ARO**	**AS**	**Pi2**	1.50	21.41	1.85	4.78	41.76	1.20	8.09	15.89	0.03
**ARO**	**AS**	**Pi1**	1.75	21.84	1.49	4.70	41.47	0.83	8.51	15.26	0.04
**ARO**	**AS**	**Pi0.5**	1.60	20.87	1.48	2.34	40.44	1.49	8.30	20.12	0.04
**ARO**	**AS**	**Pi0.25**	1.50	18.14	1.67	1.76	41.93	1.94	8.05	21.22	0.05

			C14:0	C16:0	C16:1	C18:0	C18:1n9c	C18:2n6t	C18:2n6c	C18:3n6	C20:4n6
**MAL**	**YE**	**Pi4**	0.96	16.01	0.14	13.54	11.18	0.09	9.96	4.27	37.75
**MAL**	**YE**	**Pi2**	0.57	12.02	0.10	13.86	9.40	0.09	9.75	4.42	43.54
**MAL**	**YE**	**Pi1**	0.61	12.02	0.10	12.65	8.68	0.08	8.72	4.99	45.58
**MAL**	**YE**	**Pi0.5**	0.76	13.95	0.12	12.52	8.22	0.08	7.77	5.69	45.07
**MAL**	**YE**	**Pi0.25**	0.46	10.73	0.08	13.35	8.61	0.07	8.45	4.47	47.00
**MAL**	**AS**	**Pi4**	1.87	18.60	0.16	13.50	10.86	0.18	8.23	4.26	34.30
**MAL**	**AS**	**Pi2**	1.72	17.00	0.19	14.13	15.84	0.07	7.88	4.28	27.12
**MAL**	**AS**	**Pi1**	2.05	23.73	0.26	13.10	23.25	0.05	6.07	2.41	17.47
**MAL**	**AS**	**Pi0.5**	1.73	21.98	0.28	11.53	25.07	0.11	5.13	1.69	15.57
**MAL**	**AS**	**Pi0.25**	2.06	24.52	0.41	10.34	24.53	0.36	3.53	1.39	8.75
